# Effectiveness and Related Factors of Narrative Messages in Correcting Health-Related Misinformation: Protocol for a Systematic Review

**DOI:** 10.2196/69414

**Published:** 2025-09-24

**Authors:** Tsuyoshi Okuhara, Hiroko Okada, Rie Yokota

**Affiliations:** 1 Department of Health Communication School of Public Health The University of Tokyo Tokyo Japan; 2 Department of Medical Communication School of Pharmacy and Pharmaceutical Sciences Hoshi University Tokyo Japan

**Keywords:** internet, social media, social networks, misinformation, false information, infodemics, infodemiology, debunking, prebunking, correction, refutation, narrative, story, health information, health communication

## Abstract

**Background:**

The internet and social media have become essential sources of health information for patients and citizens; however, they often disseminate misinformation that lacks scientific evidence. Health-related misinformation can undermine evidence-based treatment, weaken patient-provider relationships, and contribute to adverse health outcomes. Although narratives have been proposed as a promising approach to countering misinformation, their effectiveness remains inconsistent and influenced by various factors.

**Objective:**

The aim of this study is to assess the effectiveness of narrative messages in correcting health-related misinformation compared to nonnarrative messages. It also seeks to identify message-, sender-, and recipient-related factors that influence the effectiveness of narrative-based corrections.

**Methods:**

This systematic review will follow the PRISMA (Preferred Reporting Items for Systematic Reviews and Meta-Analyses) guidelines. Comprehensive searches will be conducted across databases, including PubMed, MEDLINE, CINAHL, PsycINFO, and Web of Science, using keywords related to narratives and correction of health-related misinformation. This review will include quantitative studies evaluating narrative-based corrections for health-related misinformation in experimental and quasi-experimental studies. Studies unrelated to health misinformation or where the full text is unavailable will be excluded. No restrictions on publication year will apply. Only papers written in English will be included. Two independent reviewers will screen the papers using Rayyan QCRI software, with disagreements resolved by a third reviewer. Data extraction will cover health topics (eg, vaccination, tobacco), study characteristics (eg, author, publication year), narrative characteristics (eg, definition of narrative, theoretical foundation), participant characteristics (eg, sociodemographic), methodology (eg, study design, content of interventions and comparators, outcomes and measures, moderating and mediating factors), main results, and discussion. The quality of the eligible studies will be assessed using the Cochrane Risk of Bias 2 tool and the Risk of Bias In Non-randomized Studies - of Interventions tool.

**Results:**

The results will be summarized in tables and presented as a descriptive review addressing the effectiveness of narrative corrections in health-related misinformation and the factors influencing their success. The implications of these results for future studies and practices will be elucidated. The findings of this review will be presented at a relevant conference and submitted to a peer-reviewed journal for publication. The aim is to complete the submission process by the northern summer of 2025.

**Conclusions:**

Narrative messages represent a theoretically promising strategy for countering health-related misinformation; however, their effectiveness is context-dependent. This review will offer critical insights into the factors that influence the success of narrative corrections for health-related misinformation, contributing to the development of improved correction strategies and a theoretical understanding of narrative corrections.

**International Registered Report Identifier (IRRID):**

DERR1-10.2196/69414

## Introduction

The internet and social media are the major sources for individuals seeking health information, regardless of their age [1,2]. However, these platforms are often rife with health-related misinformation that lacks scientific evidence [3]. Compared to accurate health information, misinformation is more likely to reach a wider audience and maintain its influence over time [4]. This misinformation disrupts appropriate health behaviors and adherence to evidence-based treatments [5], undermines the relationship between patients and caregivers and health care providers [6], and contributes to an increased risk of adverse health outcomes, including mortality [7,8]. To address these challenges, previous studies have focused on developing and evaluating effective strategies to correct health-related misinformation and mitigate its negative effects [9,10].

Many studies have evaluated the effectiveness of corrective strategies by providing factual and didactic explanations highlighting the inaccuracies and logical fallacies of misinformation [9]. However, the findings of these studies are mixed, with outcomes ranging from the successful resolution of misperceptions to the persistence or exacerbation of false beliefs [11]. Researchers have identified several factors that complicate the correction of health-related misinformation. A key factor is the mental models that individuals construct to understand causal relationships among events [12]. These mental models serve as frameworks for interpreting information but are often resistant to change. Individuals are reluctant to discard the presumed causal explanations embedded in their established mental models [13]. Consequently, fact-based corrections alone can be insufficient to provide alternative explanations that override preexisting beliefs, ultimately failing to shift mental models grounded in misinformation [14].

In this review, we define correction as an intentional communication effort to modify beliefs, attitudes, or behavioral intentions that have been shaped by exposure to misinformation. This concept differs from disclosure, which often involves the provision of clarifying or supplementary information—such as price comparisons or risk statements—without directly addressing or aiming to undo prior misperceptions. Prior studies, particularly in the context of prescription drug advertising, have shown that while disclosures may enhance transparency, they do not always lead to correction of misperceptions [15,16]. Our review focuses specifically on corrections that aim to alter misinformation-driven mental models or beliefs.

Researchers have used narratives as a corrective tool. Narratives are defined as messages that depict “connected events and characters that have an identifiable structure, are bounded in space and time, and contain implicit or explicit messages about the topic being addressed” [17]. Previous studies [18-23] have identified several persuasive mechanisms within narratives that make them particularly effective in addressing misinformation. Narratives serve as the fundamental means for individuals to interpret and understand the world. By linking the actions and outcomes experienced by characters in a cause-and-effect framework, narratives can provide a compelling mental model for explaining interconnected events and their underlying causal relationships [18]. The concept of narrative transportation [19], which refers to the immersion of an individual into a story and the ability to envision themselves within events, allows for the mental simulation of the alternative model introduced in the narrative [20]. Consequently, narrative-based corrections have the potential to supplant the existing mental models shaped by exposure to misinformation [21,22]. In addition, narratives are often more persuasive because of their inherent simplicity and accessibility, making them easier to process than argument-based messages [23]. Moreover, narratives are often perceived as having less overt persuasive intent, which reduces audience resistance and minimizes counterarguments [24,25].

Although narrative-based corrections are theoretically promising, empirical evidence regarding their effectiveness has been inconsistent [26]. For instance, one study demonstrated that a narrative-based correction message significantly reduced counterarguments and increased positive beliefs regarding nicotine replacement therapy compared to a nonnarrative correction message [27]. In contrast, another study [28] found no significant difference in the effects of narrative versus nonnarrative corrections on the intentions of participants to vaccinate their children with the measles, mumps, and rubella vaccine.

These conflicting findings suggest that the effectiveness of narrative corrections in combating misinformation may be influenced by a variety of factors, including those related to the message, sender, and recipient. For example, one study explored how emotional language and an emotionally impactful ending could moderate the effectiveness of narrative correction messages [29]. Another study examined the effects of a refutational ending, which explicitly highlights the correction of narrative messages [30]. Regarding sender-related factors, one study investigated the mediating role of the perceived credibility of the information source [28]. Another study focused on the moderating effect of the perceived relational closeness between the sender and recipient of a message [27]. Regarding recipient-related factors, one study explored how the need for cognition of the message recipients moderates the effectiveness of narrative corrections [31]. Another study examined the moderating effect of the motivation of recipients to use social media [32].

Thus, the inconsistencies in the effectiveness of narrative corrections for health-related misinformation observed in previous studies suggest that multiple factors contribute to success. This systematic review aims to synthesize existing studies on the effectiveness of narrative messages in correcting health-related misinformation and to identify the various factors that affect their effectiveness. The findings from this review will inform future research and practical applications in the field of health communication. The research questions addressed in this review are as follows.

1. Are narrative messages more effective than nonnarrative messages in correcting health-related misinformation?

2. What message-related factors enhance the effectiveness of narrative messages in correcting health-related misinformation?

3. What sender- and recipient-related factors moderate or mediate the effectiveness of narrative messages in correcting health-related misinformation?

## Methods

### Overview

This systematic review will be conducted in accordance with the guidelines provided in the PRISMA (Preferred Reporting Items for Systematic Reviews and Meta-Analyses) statement [33] (Multimedia Appendix 1). This protocol will be registered with PROSPERO after peer review. The literature search had already commenced at the time of acceptance, and the analysis is planned to be completed by March 30, 2025.

### Literature Search

Searches will be conducted across several databases, including PubMed, MEDLINE, CINAHL, PsycINFO, and Web of Science. A combination of keywords, informed by previous studies [34,35], will be used to search the abstracts within these databases: (narrative OR story OR storytelling OR testimonial OR anecdote OR exemplar OR episode OR vignette OR experience) AND (debunk* OR prebunk* OR correct* OR refut* OR rebuttal* OR counter* OR combat* OR retract* OR belief correction OR misinformation rejection) AND (misinformation OR disinformation OR misperception OR mislead* OR misbelief* OR fake OR conspiracy* OR myth* OR rumo* OR fals* OR fraud* OR scam* OR wrong OR inaccurate OR incorrect OR gossip OR hoax* OR fallac* OR infodemic OR misperception OR misconception OR suspicion* OR skeptic* OR unverified OR unscientific OR lie OR anti-vaccin* OR anti-vax* OR anti-scien*). No restrictions will be applied to the publication year during the searches. Additionally, the reference lists of the relevant studies will be reviewed to identify other potentially eligible publications.

### Eligibility Criteria

Intervention studies that quantitatively evaluated the impact of narrative-based corrections for health-related misinformation will be included. For this systematic review, misinformation will be defined as “information that asserts or implies claims that are inconsistent with the weight of accepted scientific evidence at the time” [36], following the definition proposed in the 2025 Consensus Study by the US National Academies of Sciences, Engineering, and Medicine. This definition emphasizes the scientific status of misinformation, irrespective of the intent behind its dissemination, and provides a consistent foundation for evaluating studies included in this review. Adopting the World Health Organization’s definition of health as “a state of complete physical, mental, and social well-being and not merely the absence of disease or infirmity” [37], a comprehensive interpretation of health-related misinformation will be applied. Therefore, this review will cover topics related to health, such as physical well-being (eg, infectious diseases, obesity, reproductive health) as well as mental and social well-being (eg, nutrition, developmental health); relevant literature in these areas will be included. The eligibility criteria are summarized in Table S1 of Multimedia Appendix 2.

### Study Selection

The Rayyan QCRI software [38] will be used for the screening process. Duplicate entries will be automatically identified and removed by this tool. Initially, the titles and abstracts of the studies will be reviewed to determine eligibility based on the selection criteria. The first author (TO) and the second author (HO) will independently assess the titles and abstracts. Any disagreements between the reviewers will be resolved through discussion until a consensus is reached. In cases where consensus cannot be reached, the third author (RY) will be consulted to assist in resolving the issue. Subsequently, the full texts of papers that meet the initial inclusion criteria will be reviewed independently by the first (TO) and second (HO) reviewers. If there are disagreements, the third reviewer (RY) will facilitate resolution through discussion. The screening procedure will be presented using a PRISMA flow diagram.

### Data Extraction and Synthesis

The first reviewer (TO) will extract descriptive information from the eligible studies into Microsoft Excel, and the second reviewer (RY) will review the extracted data to identify errors. The data to be extracted are outlined in Table S1 of Multimedia Appendix 3. The outcomes to be extracted will include distinct constructs reflecting the effects of corrections, including (1) belief change (eg, shifts in factual accuracy judgments), (2) attitude change (eg, evaluations of health behaviors or interventions), (3) behavioral intention (eg, willingness to vaccinate), and (4) misinformation rejection (eg, explicit rejection or skepticism toward misinformation), following previous studies that differentiate these constructs as separate outcome categories [39-41].

The results will be summarized in a compact table and synthesized through a descriptive and narrative review. Given the anticipated heterogeneity in study designs, narrative intervention formats (eg, testimonial, anecdote, story, vignette), outcome measures (eg, belief change, attitude, behavioral intention), and types of comparators (eg, non-narrative text, factual correction, or control), this review will not conduct a meta-analysis. The methodological and conceptual diversity across studies is expected to preclude meaningful effect size pooling. Instead, a narrative and descriptive synthesis will be conducted to explore patterns in the effectiveness of narrative corrections and to address the predefined research questions, focusing on message-, sender-, and recipient-related factors. The findings will be discussed along with their implications for future research and practice as each of the research questions is addressed.

### Quality Assessment

To evaluate the methodological quality of the eligible studies, the first reviewer (TO) will apply the Cochrane Risk of Bias 2 (RoB-2) tool for assessing the risk of bias in randomized controlled trials [42]. This tool allows for the assessment of the overall risk of bias in each study by examining areas such as randomization processes, allocation concealment, handling of missing data, outcome measurement, and outcome reporting. Each of these domains will be categorized as low risk, some concerns, or high risk based on the responses to several signaling questions. For nonrandomized studies, the Risk Of Bias In Non-randomized Studies - of Interventions (ROBINS-I) tool will be applied to ensure appropriate assessment of methodological quality [43]. The second reviewer (HO) will verify the quality assessment results by using the RoB-2 tool and ROBINS-I tool, and a third reviewer (RY) will be consulted if required. The results of the quality assessment will not be used as a basis for excluding studies from the review.

## Results

The findings will be summarized in tables and presented as a descriptive review. The implications of the results for future research and practices will be explored while addressing the research questions. The results of this review will be shared at an appropriate conference and submitted for publication in a peer-reviewed journal. It is anticipated that the review will be submitted for publication by the northern summer of 2025.

## Discussion

### Principal Findings

This systematic review aims to be the first comprehensive examination of previous studies comparing the effectiveness of narrative and nonnarrative approaches in correcting health-related misinformation. However, a simple comparison between these 2 approaches is insufficient. Although the use of narratives to correct health-related misinformation holds theoretical promise, the results of previous studies have been inconsistent [26]. This inconsistency is likely to be due to various factors that influence the effectiveness of narratives. Previous studies have explored message-related factors that may affect narrative effectiveness, such as the inclusion of a refutational ending [30] and conversion narratives [32], in which the narrator undergoes a change in belief. This review will examine the types and effects of these message-related factors. Additionally, studies have identified factors that moderate or mediate the effectiveness of narratives in correcting health-related misinformation. These include sender-related factors such as source credibility [28] and the type of endorser [27] and recipient-related factors, including motivations for using social media [32] and issue involvement [30]. This systematic review will provide insights into the types and effects of moderating and mediating factors on the effectiveness of narrative corrections. In summary, this review will be a comprehensive overview of the effectiveness of narrative interventions and the various factors influencing their success in correcting health-related misinformation, thereby contributing to the development of narrative persuasion theories and more effective future correction strategies.

### Limitations

This systematic review will have limitations. Despite employing a comprehensive search strategy and using search terms based on previous systematic reviews, the possibility of incomplete retrieval of relevant literature cannot be entirely excluded. In addition, this review may overlook relevant studies published in languages other than English. Although a quality assessment of the studies included will be conducted, the findings of this review may still be influenced by the risk of biases present in the studies. Moreover, the limitations inherent in the qualitative synthesis of evidence as opposed to employing meta-analytical techniques should also be recognized.

### Conclusion

Over the past few decades, the infrastructure for communication technologies has become increasingly complex, with social media platforms facilitating the easy dissemination of accurate and inaccurate information. Addressing health-related misinformation has become a critical concern in medicine and public health. This systematic review will examine the potential of narratives as a theoretically promising approach to correcting health-related misinformation. It will provide a comprehensive overview of the effectiveness of narrative-based interventions and explore the various message-, sender-, and recipient-related factors that may influence their success. The synthesis of these findings will contribute to theoretical advances in future research on the use of narratives to combat health-related misinformation and inform more effective corrective practices.

## Appendix

Multimedia Appendix 1 PRISMA checklist.

Multimedia Appendix 2 Eligibility criteria.

Multimedia Appendix 3 Data extraction.

## Abbreviations

PRISMA: Preferred Reporting Items for Systematic Reviews and Meta-Analyses
RoB-2: Cochrane Risk of Bias 2
ROBINS-I: Risk Of Bias In Non-randomized Studies - of Interventions
