# Supplementary material for: Effectiveness and Related Factors of Narrative Messages in Correcting Health-Related Misinformation: Protocol for a Systematic Review
Source: JMIR Res Protoc. 2025 Sep 24;14:e69414. doi: 10.2196/69414 (PMC12508667; doi:10.2196/69414)
Supplement: Multimedia Appendix 2 [file resprot_v14i1e69414_app2.doc]

Multimedia Appendix 2

| **Table S1. Eligibility criteria** |
| --- |
| **Inclusion criteria** |
| - Studies focused on the correction of health-related misinformation using narratives. - All quantitative study designs will be considered, including experimental (e.g., randomized controlled trials, quasi-randomized controlled trials, and non-randomized trials) and quasi-experimental research (e.g., pre-test-post-test designs and post-test-only designs). - Only quantitative studies (e.g., experimental and quasi-experimental) evaluating the effectiveness of narrative-based corrections will be included. - Studies involving narrative-based interventions. Any form of comparator (e.g., interventions other than narrative). Studies without a comparator. - Studies measuring outcomes, such as beliefs, perceptions, behavioral intentions, and attitudes. - Studies involving participants of any age, sex, ethnicity, and nationality. - Grey literature (e.g., conference proceedings) if it contains sufficient information for assessment of eligibility. - Any publication year. - Studies published in English. |
| **Exclusion criteria** |
| - Studies that do not align with the objectives of the review (i.e., studies unrelated to narrative-based correction of misinformation). - Qualitative studies and literature reviews will be excluded. - Studies focusing on misinformation outside the health domain (e.g., political misinformation). - Studies that are not available in full-text format. - Papers written in languages other than English. |
