# Supplementary material for: Effectiveness and Related Factors of Narrative Messages in Correcting Health-Related Misinformation: Protocol for a Systematic Review
Source: JMIR Res Protoc. 2025 Sep 24;14:e69414. doi: 10.2196/69414 (PMC12508667; doi:10.2196/69414)
Supplement: Multimedia Appendix 3 [file resprot_v14i1e69414_app3.doc]

**Multimedia Appendix 3**

| **Table S1. Data extraction** |
| --- |
| - Health topic (e.g., vaccination and tobacco) - Study characteristics (author, year of publication, type of paper, and country) - Characteristics of narrative (definition of narrative, theoretical foundation, type of narrative, narrator, length of narrative, type of media, and other information about narrative) - Participant characteristics (number of participants, sex, age, and other demographic information including generalizability, such as student or non-student) - Methodology (study design, sample size of each group, content of exposures, such as interventions and comparators, outcomes and measures, message-related factors, sender-related factors, recipient-related factors, moderating factors, and mediating factors) - Main results and discussion |
